# Dzyaloshinskii-Moriya Interaction and Spiral Order in Spin-orbit Coupled Optical Lattices

**DOI:** 10.1038/srep10050

**Published:** 2015-05-27

**Authors:** Ming Gong, Yinyin Qian, Mi Yan, V. W. Scarola, Chuanwei Zhang

**Affiliations:** 1 Department of Physics, the University of Texas at Dallas, Richardson, Texas, 75080 USA; 2 Department of Physics and Center for Quantum Coherence, The Chinese University of Hong Kong, Shatin, N.T., Hong Kong, China; 3 Department of Physics, Virginia Tech, Blacksburg, Virginia 24061 USA

## Abstract

We show that the recent experimental realization of spin-orbit coupling in ultracold atomic gases can be used to study different types of spin spiral order and resulting multiferroic effects. Spin-orbit coupling in optical lattices can give rise to the Dzyaloshinskii-Moriya (DM) spin interaction which is essential for spin spiral order. By taking into account spin-orbit coupling and an external Zeeman field, we derive an effective spin model in the Mott insulator regime at half filling and demonstrate that the DM interaction in optical lattices can be made extremely strong with realistic experimental parameters. The rich finite temperature phase diagrams of the effective spin models for fermions and bosons are obtained via classical Monte Carlo simulations.

The interplay between ferroelectric and ferromagnetic order in complex multiferroic materials presents a set of compelling fundamental condensed matter physics problems with potential multifunctional device applications^1^,^2^,^3^,^4^. Ferroelectric and ferromagnetic order compete and normally cannot exist simultaneously in conventional materials. While in some strongly correlated materials, such as the perovskite transition metal oxides^5^,^6^,^7^,^8^,^9^,^10^, these two phenomena can occur simultaneously due to strong correlation. Nowadays construction and design of high-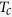 magnetic ferroelectrics is still an open and active area of research^11^. These materials incorporate different types of interactions, including electron-electron interactions, electron-phonon interactions, spin-orbit (SO) couplings, lattice defects, and disorder, making the determination of multiferroic mechanisms a remarkable challenge for most materials^12^,^13^. In this context an unbiased and direct method to explore multiferroic behavior in an ideal setting is highly appealing.

On the other hand, the realization of a superfluid to Mott insulator transition of ultracold atoms in optical lattices^14^ opens fascinating prospects^15^ for the emulation of a large variety of novel magnetic states^16^,^17^,^18^ and other strongly correlated phases found in solids because of the high controllability and the lack of disorder in optical lattices. For instance, it has been shown^16^,^17^ that the effective Hamiltonian of spin-1/2 atoms in optical lattices is the XXZ Heisenberg model in the deep Mott insulator regime. On the experimental side, superexchange interactions between two neighboring sites have already been demonstrated^19^ and quantum simulation of frustrated classical magnetism in triangular optical lattices has also been realized^20^. These experimental achievements mark the first steps towards the quantum simulation of possible magnetic phase transitions in optical lattices.

In this paper, we show that the power of optical lattice systems to emulate magnetism can be combined with recent experimental developments^21^,^22^,^23^,^24^ realizing SO coupling to emulate multiferroic behavior. Recently, SO coupled optical lattices have been realized in experiments for both bosons^25^ and fermions^26^, where interesting phenomena such as flat band^26^,^27^,^28^ can be observed. The main findings of this work are the following: (I) We incorporate spin-orbit and Zeeman coupling into an effective Hamiltonian for spin-1/2 fermions and bosons in optical lattices in the large interaction limit. We show that SO coupling leads to an effective in-plane Dzyaloshinskii-Moriya (DM) term, an essential ingredient in models of spiral order and multiferroic effects in general. The DM term is of the same order as the Heisenberg coupling constant. (II) We study the finite temperature phase diagram of the effective spin model using classical Monte Carlo (MC). We find that competing types of spiral order depend strongly on both SO and effective Zeeman coupling strength. (III) We find that the critical temperature for the spiral order can be of the same order as the Heisenberg coupling constant. Thus, if magnetic quantum phase transitions can be emulated in optical lattices, then spiral order and multiferroic-based models can also be realized in the same setup with the inclusion of SO coupling.

## Results

### Effective Hamiltonian

We consider spin-1/2 ultracold atoms loaded into a two-dimensional (2D) square optical lattice. We restrict ourselves to the deep Mott insulator regime where the charge/mass degree of freedom is frozen while the spin degree of freedom remains active. Here the atomic hyperfine levels map onto effective spin states. The scattering length between atoms in optical lattices can be controlled by a Feshbach resonance. Certain atoms, e.g., ^40^K, exhibit considerable tunability^29^. To derive the inter-spin interaction in this regime we first consider a two-site tight-binding model,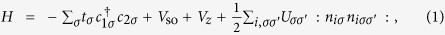 where 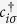 creates a particle (either a boson or a fermion) in a Wannier state, 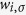, localized at a site 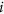 and in a spin state 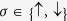. 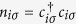 is the number operator. The tunneling and interaction matrix elements are 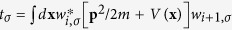 and 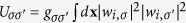, respectively, where 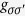 is the interaction strength between species 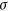 and 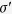, 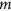 is the mass of the atom, and 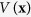 is a lattice potential. Here 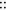 denotes normal ordering. For a general theory the tunneling is assumed to be spin dependent, which is a feature unique to ultracold atom systems^17^,^18^. The second term is the Rashba SO coupling^30^, written in the continuum as 
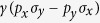
. But on a lattice it can be written as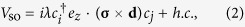 where 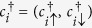, 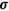 denotes Pauli matrices, and 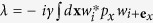 is the SO coupling strength. 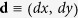 is the vector from a site at position 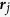 to a site at 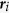, where 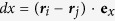 and 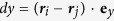. Eq. 2 describes the tunneling between neighboring sites paired with a spin flip. The magnitude and sign of 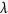 can be tuned in experiments using coherent destructive tunneling methods^31^. The third term is the external Zeeman field 
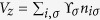
 with 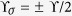.

In the deep Mott insulator regime, the degeneracy in spin configurations is lifted by second order virtual processes. The effective Hamiltonian 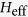 can be obtained using perturbation theory. We take the Mott insulator as the unperturbed state and derive the corrections of the effective Hamiltonian by the standard Schrieffer-Wolf transformation^17^,^32^. The Schrieffer-Wolf transformation applies a canonical transformation 
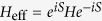
 to obtain the second order Hamiltonian 
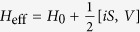
 by eliminating the first order term using 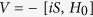. In the spin representation we define 
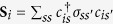
, and extend the two-site model to the whole lattice, yielding
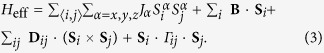


**Figure 1:**
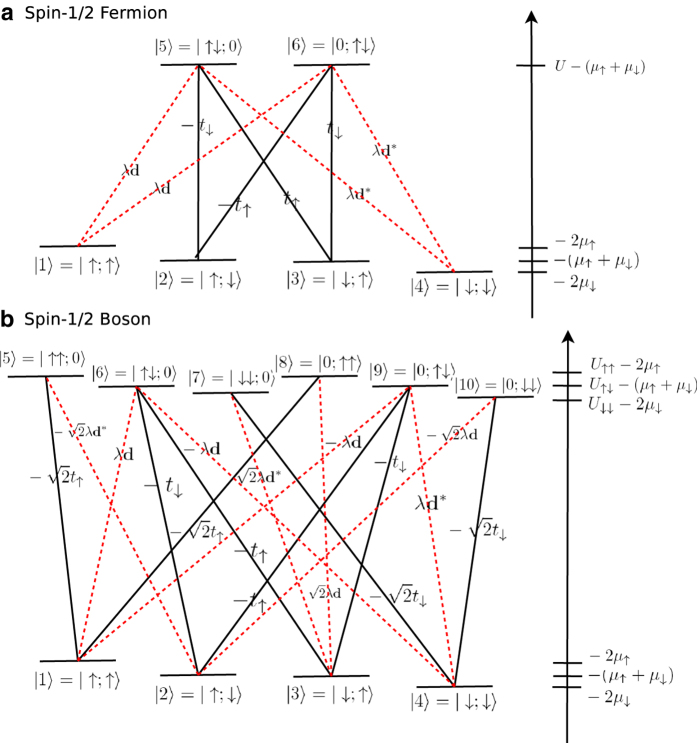
Transition processes due to different tunneling mechanisms. Spin-conserving tunneling (solid lines, 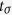 terms) and SO coupling mediated tunneling (dashed lines, 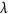 terms) are plotted for spin-1/2 fermions (**a**) and spin-1/2 bosons (**b**) 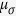 is the chemical potential. The lowest 4 levels are ground states, and the higher energy levels are the excited states.

The first two terms are Heisenberg exchange and Zeeman terms, respectively, while the last two terms arise from SO coupling. In solid state systems the third term is called the DM interaction^33^,^34^, which is believed to drive multiferroic behavior. The definition of the D vector and the Γ tensor will be presented below. The structure of these terms can be derived from basic symmetry analyses but the coefficients must be computed microscopically. In the following we derive the coefficients in Eq. 3 by considering the coupling between four internal degenerate ground states 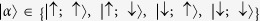 through the spin independent and dependent tunnelings 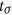 and 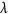. The couplings are different for fermions and bosons, as illustrated in Fig. 1.

### Fermionic atoms

For fermionic atoms, there are only two possible excited states 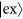 = 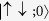 and 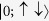, as shown schematically in Fig. 1(a). We find 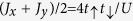, 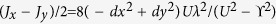, and 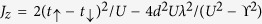, with 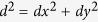. The DM interaction coefficient is 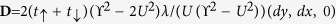, and the effective Zeeman field contains 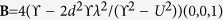. Note that without SO coupling the model reduces to the well-known XXZ Heisenberg model with rotational symmetry^16^,^17^. However, this symmetry is broken by the SO coupling, yielding an XYZ-type Heisenberg model. Similar results are also observed for bosons.

### Bosonic atoms

For bosonic atoms, there are six excited states 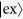 = 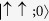, 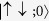, 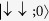,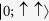, 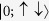, 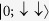, as shown in Fig. 1(b). Without SO coupling, the only allowed inter-state second-order transition is between 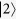 and 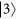, similar to the fermionic case. The presence of SO coupling permits other inter-state transitions, therefore the bosonic case is much more complex than the fermionic case. For simplicity we only show the results for 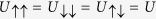, which yields 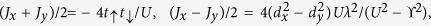 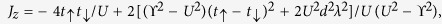 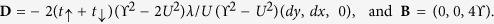


The last term in Eq. 3 reads as 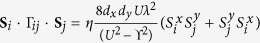, where 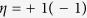 for fermions (bosons). This term arises from the coupling between states 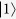 and 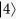, 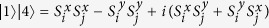. Here the real part contributes asymmetric terms to the Heisenberg model, while the imaginary part contributes to Γ_*ij*_. In a square lattice with 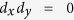, this term vanishes. However, for tilted lattices, such as triangular and honeycomb, this term should be significant.

### Lattice parameters

We estimate the possible parameters that can be achieved in a square optical lattice 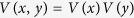, where 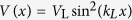. We define the lattice depth 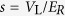 in units of he recoil energy 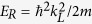, where 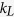 is the wavevector of the laser. The SO coupling coefficient is given by 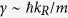, 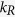 is the wavevector of the external Raman lasers, and 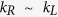 in most cases. The Raman lasers are pure plane waves, and serve as a perturbation to the hopping between adjacent sites.

**Figure 2:**
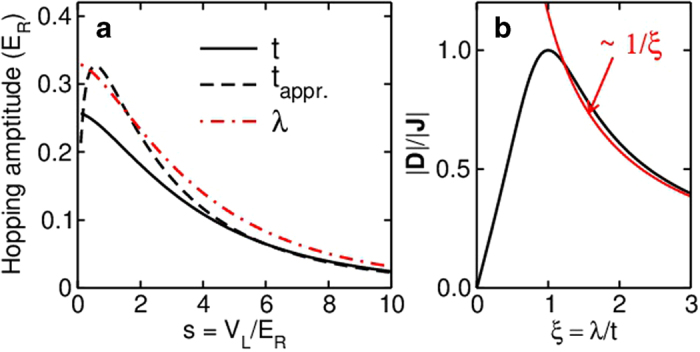
Tunable parameters in an optical lattice. (**a**) Tunneling amplitudes as a function of lattice depth. 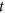 is the hopping due to the kinetic energy, 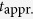 is the analytic expression derived in the deep lattice regime, and 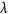 is the SO mediated hopping strength. (**b**) Plot of 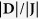 as a function of 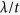 for 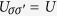, 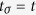.

We use the Wannier functions of the lowest band without SO coupling to calculate the tight binding parameters 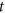 and 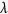. In a square lattice, coordinates decouple and the Bloch functions are Mathieu functions. The Wannier functions can be obtained from the Fourier transform of the Bloch functions. Our numerical results are presented in Fig. 2(a). The large 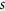 limit, 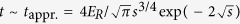, is also plotted for comparison. Note that 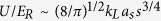 is in general much larger than 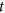 and can be controlled through a Feshbach resonance independently.

In Fig. 2(b) we plot 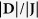 as a function of 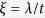 for 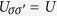, 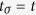. 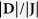 reaches the maximum value of 1.0 at 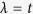. This is in sharp contrast to models of weak multiferroic effects in solids with 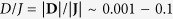, which is generally induced by small atomic displacements^35^. Optical lattices, by contrast, can be tuned to exhibit either weak or strong DM terms. This enhanced tunability enables optical lattice systems to single out the effects of strong DM interactions and study the impact of the DM term.

There are notable differences between our model and corresponding models in solids (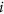) In solids the SO coupling arises from intrinsic (atomic) SO coupling and 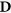 is generally along the 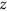 direction (out of plane). However, in our model 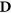 is in the plane and the out of plane component is zero. (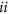) In our effective spin model, 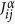 depends on the direction of the bond (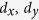) and the SO coupling strength, while in solids 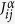 is independent of SO coupling due to its negligible role.

### Spiral order and multiferroics in 2D optical lattices

We now explore the rich phase diagrams of the effective spin Hamiltonian using classical MC simulations. Classical MC has been widely used to explore the phase diagrams of the Heisenberg model with DM interactions in the context of solids^11^,^36^,^37^,^38^ (thus weak DM interactions). This method may not be used to determine the precise boundaries between different phases but can be an efficient tool to determine different possible phases. Due to the unique features of our effective model (e.g., strong DM interactions) the phase diagrams we present here are much more rich and comprehensive than those explored in the context of solids. We focus on the regime where 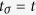, 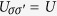 (spin independent), and 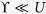, and define 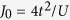 as the energy scale. The rescaled effective Hamiltonian becomes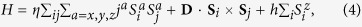 where 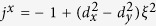, 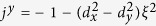, 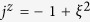, 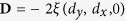, and 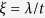.

Eq. 4 hosts a variety of magnetic and spin spiral phases, which are generally characterized by the magnetic and spiral order parameters^39^,^40^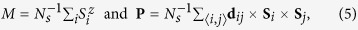 where 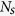 is the number of sites. However, these two order parameters do not fully characterize the phase diagrams because in some cases there are still local magnetic or spiral orders although both 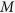 and 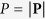 are vanishingly small. In these cases, we also take into account the spin structure factor:
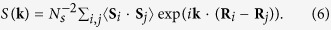

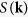
 shows peaks at different positions in momentum space for different phases. For instance, the peak of the spin structure factor is at 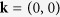 for ferromagnetic phases, 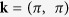 for antiferromagnetic phases, and 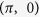 (or 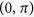) for the flux spiral phase (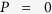 but with nontrivial local spin structure). General spiral orders correspond to other 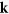. We obtain the phase diagrams by analyzing both the order parameters and spin structure factors. We have not checked for long range order in the spin structure factor. We expect quasi-long range order to accompany magnetized phases at low 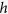, e.g., a ferromagnetic phase for 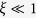.

**Figure 3:**
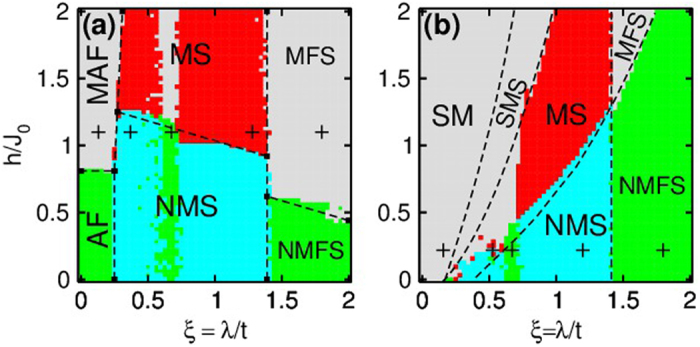
Phase diagrams of 2D optical lattices. Classical Monte Carlo simulations are performed for an 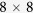 lattice with fermions (**a**) and bosons (**b**) at temperature 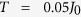. The phases diagrams are determined by the magnetization order, the spiral order, and the spin structure factor. Different regions correspond to: 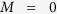, 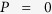 for green, 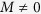, 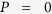 for grey, 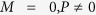 for cyan, and 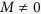, 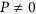 for red. The abbreviations are: (**a**) AF: antiferromagnetic phase with zero total magnetization; MAF: antiferromagnetic phase with non-zero total magnetization; NMS: zero magnetization spiral order; MS: magnetic spiral order; NMFS: nonmagnetic flux spiral phase; MFS: magnetic flux spiral phase. In (**b**) SM: simply magnetic order; SMS: simply magnetic spiral order: Other abbreviations are the same as in (**a**) The dashed lines are guides to the eye. The spin structure factors of the points marked by plus signs are shown in Fig. 4.

**Figure 4:**
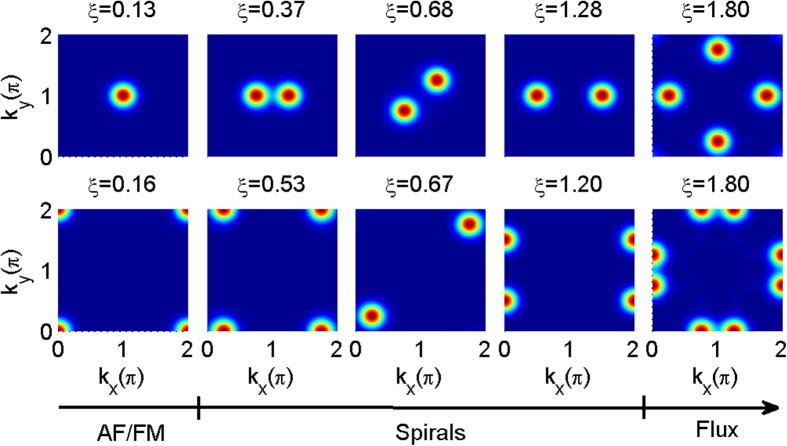
Spin structure factors for different quantum phases marked by plus signs in Fig. 3. The upper panels show the results for fermions at 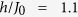, while the lower panels show the results for bosons at 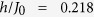.

The phase diagrams of an 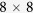 lattice in Fig. 3 show a rich interplay between magnetic orders and spin spiral orders. For instance, for fermions with small SO coupling (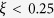), the ground states are anti-ferromagnetic states with zero (non-zero) magnetization for a Zeeman field 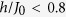 (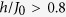). While for large SO coupling (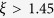), the ground states are either nonmagnetic or magnetic flux spiral phases (similar to the flux phase with a small spiral order 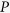). For 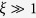 the DM term is not important because 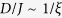, therefore the pure flux phase with zero spiral order can be observed. Similarly, the increasing SO coupling for bosonic atoms gives rise to a series of transitions from simply magnetic (ferromagnetic at small 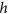) order to simply magnetic spiral order (with zero total spiral order but local spiral structure), then to magnetic spiral orders (or non-magnetic spiral orders) and finally to flux spiral orders. The emergence of the spiral order and flux order with increasing SO coupling can be clearly seen from the change of the spin structure factors in Fig. 4, which shift from 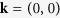 or 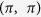 to 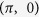 and 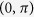.

**Figure 5:**
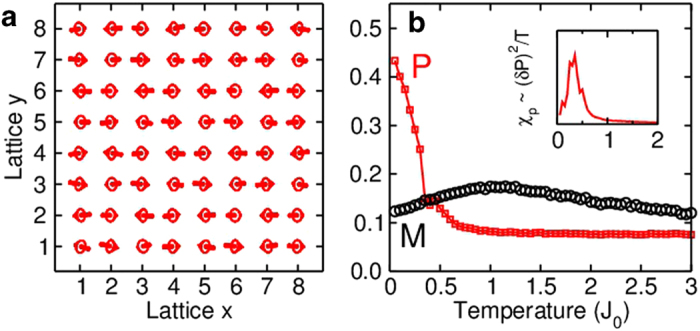
Spin configurations and phase transitions. (**a**) The spin configuration of fermions in an 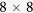 lattice at 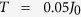, 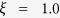 and 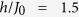. The corresponding magnetization and spiral order as a function of temperature is shown in (**b**) The inset plots 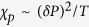 vs. temperature, which indicates a phase transition at 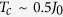. Similar features can also been found for bosons with the same parameters.

The spin spiral order phase transition temperature is comparable to the magnetic phase transition temperature, 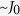. In Fig. 5(a), we plot the spin configuration of fermions at 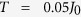, 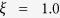 and 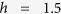 (MS phase), which shows clear spiral ordering. The corresponding order parameters 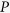 and 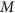 are plotted in Fig. 5(b) as a function of temperature. The inset shows the susceptibility 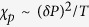. We see a phase transition at 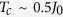, which is comparable to the magnetic critical temperature^17^ (In 2D, the Heisenberg model has a critical temperature 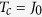 in mean-field theory). Note that spiral order can also exist in the frustrated model without SO coupling, however, the critical temperature is generally much smaller than the magnetic phase transition temperature^11^,^41^. Our results therefore show that SO coupling in the absence of frustration provides an excellent platform to search for spiral order and multiferroics-based states in optical lattices.

## Discussion

Finally we note that different spiral orders may be observed using optical Bragg scattering methods^42^, which probe different spin structure factors for different spiral orders. Similar methods have been widely used in solid state systems. Furthermore, in optical lattices, the local spin magnetization at each lattice site (thus the magnetic order 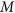) as well as the local spin-spin correlations (thus the spiral order 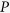) can be measured directly^43^,^44^, which provides a powerful new tool for understanding the physics of spiral orders and multiferroic effects in optical lattices.

### Note added

During the preparation of this manuscript (the initial version is available at arXiv:1205.6211) we became aware of work^45^,^46^,^47^ on similar topics.

## Methods

The phase diagrams of an 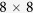 lattice are computed by classical MC methods for both fermions and bosons. The results are obtained after 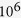 thermalization steps followed by 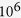 sampling steps in each MC run at low temperature (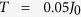). We have checked that for lower temperatures the phase diagrams do not change quantitatively. We also verify that similar phase diagrams can be obtained for larger system sizes, however, the spiral orders in a larger optical lattice become more complicated, and the boundary between different quantum phases is shifted.

## Additional Information

**How to cite this article**: Gong, M. *et al*. Dzyaloshinskii-Moriya Interaction and Spiral Order in Spin-orbit Coupled Optical Lattices. *Sci. Rep*. **5**, 10050; doi: 10.1038/srep10050 (2015).
